# Spontaneously Regressing Neonatal Oral Aphthous Ulceration of the Palate

**DOI:** 10.1155/2021/6660302

**Published:** 2021-02-06

**Authors:** Sandara Wayangi Madurapperuma, Andra Hennadige Heshan Malinga Jayaweera, Ruwan Duminda Jayasinghe

**Affiliations:** ^1^Division of Oral Medicine, Department of Oral Medicine and Periodontology, Faculty of Dental Sciences, University of Peradeniya, Peradeniya, Sri Lanka; ^2^Department of Paediatrics, Faculty of Medicine, University of Peradeniya, Peradeniya, Sri Lanka; ^3^Department of Oral Medicine and Periodontology and Centre for Research in Oral Cancer, Faculty of Dental Sciences, University of Peradeniya, Peradeniya, Sri Lanka

## Abstract

**Background:**

Neonatal oral aphthous ulceration of the palate also known as Bednar's aphthae is not an uncommon presentation. They clinically present as spontaneously regressing, shallow, and symmetrical ulcers on the posterior palate of newborns from 2 days up to 6 weeks of age. *Case Presentation*. We, herein, report a case of a one-month-old baby girl who presented with an ulcer in the posterior palate and intermittent mild fever. The patient was admitted and monitored in the ward. Haematologic investigations disclosed features of ongoing infection. Nasogastric feeding was commenced to avoid any irritation of the ulcer, and glycerine was applied on the ulcer. Antibiotic therapy was continued because of the intermittent mild fever. The lesion healed spontaneously within one week, and fever subsided afterwards. Currently, the patient is faring healthily without any complications.

**Conclusion:**

Although Bednar's aphthae is not a rare presentation, clinicians are often met with a diagnostic dilemma due to the alarming clinical presentation of this condition. Therefore, it leads to overinvestigation and overtreatment. With this case report, we would like to highlight the importance of being aware of this condition to provide the patients with the appropriate treatment.

## 1. Introduction

Aphthous stomatitis or oral aphthous ulceration is considered to be a multifactorial, idiopathic condition, affecting about 20% of the general population. Factors such as local trauma, stress, allergy, and other changes in the oral microbiome are considered as predisposing factors for this condition. Also, aphthous like ulcers are known to occur as a manifestation of underlying systemic conditions such as nutritional deficiency, Behcet's syndrome, systemic lupus erythematosus, reactive arthritis, or inflammatory bowel disease [1]. This condition is mostly seen in the adolescence and the adulthood. The occurrence of aphthous ulcers in neonates is comparatively rare [2].

Although oral ulceration is a common cause for paediatric consultation, definitive diagnosis can pose a challenge to the clinician at times. While ulcers with an infectious origin such as herpetic gingivostomatitis, herpangina, candidiasis, and hand-mouth-foot disease are fairly easy to diagnose based on the clinical picture, ulcers of traumatic and idiopathic origin often turn out to be a diagnostic dilemma [3].

However, Bednar's aphthae, also known as “ulcera pterygoidea,” was first described in 1850 by an Austrian paediatrician named Alois Bednar. They are common, spontaneously regressing, shallow, and symmetrical ulcers typically presenting on the posterior boundary of the hard palate in newborn infants from 2 days up to 6 weeks of age. Despite being fairly common, they often remain undiagnosed or misdiagnosed [3–5]. The physiopathology of Bednar' aphthae is unknown. But the traumatic action of the nipple or feeding bottle is considered a causative factor [6].

Herein, we report a case of Bednar's ulcer with some atypical features which culminated into a diagnostic dilemma with the objective of documenting some information regarding this condition which has been seldom reported in literature.

## 2. Case Presentation

A one-month-old baby girl was referred to the Division of Oral Medicine, Dental (Teaching) Hospital, Peradeniya, by the Paediatric clinic of Teaching Hospital, Peradeniya, whose mother had noticed a developing wound in the baby's mouth and complained that the baby was having fever on and off during the past two days.

**Figure 1:**
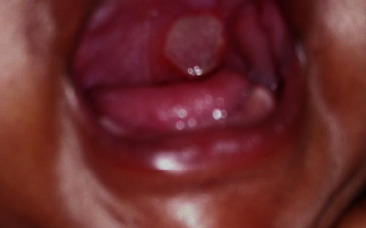
Appearance of the ulcer on the day of first visit.

The patient was alert and responsive at the time of assessment. She was medically fit and feeding well. Vital parameters were stable. Upon examination, a unilateral symmetrical ulcer with a yellowish floor and an erythematous margin, measuring approximately about 2 × 2 cm, was observed in the junction of the hard and soft palate (Figure 1).

Considering the alarming clinical appearance of the lesion and the history of fever, the patient was admitted in order to monitor the patient closely. And, empirical antibiotic treatment was commenced immediately as the presence of fever is suggestive of ongoing infection.

With the clinical findings, it was possible to arrive at the differential diagnoses of Bednar's aphthae, traumatic ulcer, osteomyelitis, and infectious disease such as tuberculosis, herpes viral infection, or candidiasis. Also, the possibility of two coexisting pathologies could not be excluded with the available information at the time.

Haematological investigations were carried out revealing that the baby was having a neutrophilic leucocytosis indicating the possibility of an ongoing infection. Moreover, the C-reactive protein level was also marginally high. However, swab, blood, and urine cultures were reported negative. The patient was commenced on intravenous cefotaxime initially, but since fever was persisting and the repeat inflammatory markers showed a marginal rise, antibiotics were changed to intravenous meropenem and metronidazole.

Then, it was deduced that the present condition could be Bednar's aphthae caused due to traumatic feeding. Therefore, nasogastric feeding was commenced discontinuing oral feeding in order to prevent further trauma. Glycerine was applied on the ulcer with a cotton swab.

The patient was closely monitored in the meantime.

The antibiotic regime was continued to tackle the intermittent pyrexia due to presumed sepsis. The question whether there is ongoing meningitis coexisting with the oral ulcer was raised. Accordingly, a computed tomography of the brain was carried out to exclude the possibility which reported no abnormal findings.

Despite the persistence of low-grade fever, the appearance of the ulcer improved drastically over a period of one week. The erythematous margins disappeared, and signs of healing appeared.

On the 10th day of hospital admission, the fever dissipated, and on the 12th day, the palatal ulcer healed completely. Following two more days of monitoring, the patient was discharged.

The patient was followed up in the paediatric clinic one week later, and the child was well without any recurrence. The mother had established breast feeding, and baby had gained weight too. After two weeks postdischarge, the baby was thriving well, and the clinicians decided that no further follow-up was needed.

## 3. Discussion

The Viennese paediatrician Alois Bednar first described neonatal oral aphthous ulceration in 1850. Thus, the occurrence of shallow ulcers in a newborn's palate was named Bednar's ulceration or Bednar's aphthae. Although the specific aetiology of aphthous ulcers is unknown, they are predisposed by trauma, hormonal changes, hypersensitivity, stress, nutritional deficiencies, and tobacco consumption. Similarly, Bednar's aphthae is known to have a traumatic origin [7].

A study published in 2010 has found out that the incidence rate of Bednar's aphthae among neonates was 15.8%. Therefore, this phenomenon may not be considered as a rare occurrence among neonates. Nebgen et al. also mentioned that Bednar's aphthae is associated with spontaneous birth at term, nutrition with formula, and mucosal hyperaemia [4].

The reported case is also a spontaneous natural birth. However, the baby was not premature and was regularly feeding on mother's milk. Although at the time of admission, she was quite irritable, was feeding regularly, and did gain weight throughout her hospital stay.

The ulcer of this reported case was round in shape, unilateral, symmetrical, and about 2 × 2 cm in size approximately. It was located in the posterior one-third of the palate. It had a yellowish floor and an erythematous margin, but exudate was not observed. Erythematous margin observed could be due to hyperaemia which is known to surround lesion of this type. With microbiological investigations, we could not isolate any organisms in this case, although secondary infection is a possibility. Also, the absence of clinical features such as irregular infiltrating borders, exudates, and cervical lymphadenitis rules out the possibility of an ongoing local infection in this case.

According to Pedra et al., the large size, position in the posterior palate, symmetry of the lesions, and afebrile course are characteristics compatible with Bednar's ulcer [3]. However, in the presented case, the baby was found with frequent episodes of fever as high as 38°C. According to the NICE (National Institute for Health and Care Excellence, United Kingdom) guidelines for paediatric fever management, children under 3 months with a temperature of 38°C or higher are in a high-risk group for serious illness [8].

The duration of fever is not a predictor of the likelihood of serious illness. But they should be assessed for infections such as Kawasaki disease, bacterial meningitis, and herpes simplex [8].

Simultaneously, the baby exhibited neutrophilic leucocytosis and an increased level of C-reactive protein level, which is also strongly indicative of ongoing infection.

Generally, Bednar's aphthae regresses spontaneously without any complications [5]. Similarly, in the case described, the ulcer healed without any scarring. To the date of this reporting, there has not been any recurrence either.

The diagnosis of this pathological entity is not supported by any particular investigations. It is purely made through clinical suspicion. Similarly, there is no specific treatment procedure for the management of Bednar's aphthae. Incorrect feeding habits must be corrected, and the patient must be monitored so that an uneventful recovery is assured [9]. However, administration of intralesional triamcinolone and photobiomodulation has been mentioned in literature as treatment modalities for this condition [10].

It is important that not only dental surgeons but also medical doctors be aware of this condition simply to avoid overinvestigation and overtreatment.

## 4. Conclusion

Although neonatal aphthous ulceration or Bednar's aphthae is not uncommon, it can pose a diagnostic challenge owing to its alarming clinical appearance. Clinicians should be knowledgeable of this condition to avoid unnecessary overinvestigation and overtreatment.

## Data Availability

The data relevant for this case report (clinical records including investigations and pictures) are available in the repository of Teaching Hospital, University of Peradeniya, Sri Lanka.

## References

[1] Plewa M. C., Chatterjee K. (2019). *Aphthous Stomatitis*.

[2] Article R., Medicine O., Natah S. S. (2004). Recurrent aphthous ulcers today: a review of the growing knowledge. *International Journal of Oral and Maxillofacial Surgery*.

[3] Pedra C., Terra C. M., Ejzenberg B., Baldacci E. R., Okay Y. (1996). Oral palatine ulcers of a traumatic nature in infants: bednar’s aphthae. *International Journal of Pediatric Otorhinolaryngology*.

[4] Nebgen S., Kasper H.-U., Schäfer D., Christ H., Roth B. (2010). Bednar’s aphthae in neonates: incidence and associated factors. *Neonatology*.

[5] Nam S.-W., Ahn S. H., Shin S.-M., Jeong G. (2016). Clinical features of bednar’s aphthae in infants. *Korean Journal of Pediatrics*.

[6] Tricarico A., Molteni G., Mattioli F. (2012). Nipple trauma in infants? Bednar aphthae. *American Journal of Otolaryngology*.

[7] Tarakji B., Gazal G., Al-Maweri S. A., Azzeghaiby S. N., Alaizari N. (2015). Guideline for the diagnosis and treatment of recurrent aphthous stomatitis for dental practitioners. *Journal of International Oral Health*.

[8] NICE Guidelines (2019). *Fever In Under 5s: Assessment and Initial Management Clinical Guideline*.

[9] Theiler M., Schwieger-Briel A., Cont M., Relly C., Meyer Sauteur P. M. (2018). Bilateral palatine ulcers in a neonate: bednar’s aphthae. *Archives of Disease in Childhood*.

[10] Almeida Mariz B. A. L., Markman R. L., Arboleda L. P. A. (2020). Bednar aphthae: a series of 3 cases in newborns. *Oral Surgery, Oral Medicine, Oral Pathology, Oral Radiology*.

